# Sex inequalities in cardiovascular risk prediction

**DOI:** 10.1093/cvr/cvae123

**Published:** 2024-06-04

**Authors:** Joshua Elliott, Barbara Bodinier, Matthew Whitaker, Rin Wada, Graham Cooke, Helen Ward, Ioanna Tzoulaki, Paul Elliott, Marc Chadeau-Hyam

**Affiliations:** Department of Infectious Diseases, Faculty of Medicine, Imperial College London, London, UK; Imperial College Healthcare NHS Trust, London, UK; Department of Epidemiology and Biostatistics, School of Public Health, Imperial College London, 90 Wood Ln, London W12 0BZ, UK; National Institute for Health Research Imperial Biomedical Research Centre, Imperial College London, The Bays, Entrance, 2 S Wharf Rd, London W2 1NY, UK; Department of Epidemiology and Biostatistics, School of Public Health, Imperial College London, 90 Wood Ln, London W12 0BZ, UK; MRC Centre for Environment and Health, School of Public Health, Imperial College London, Praed Street, London W2 1NY, UK; Department of Epidemiology and Biostatistics, School of Public Health, Imperial College London, 90 Wood Ln, London W12 0BZ, UK; MRC Centre for Environment and Health, School of Public Health, Imperial College London, Praed Street, London W2 1NY, UK; Department of Epidemiology and Biostatistics, School of Public Health, Imperial College London, 90 Wood Ln, London W12 0BZ, UK; MRC Centre for Environment and Health, School of Public Health, Imperial College London, Praed Street, London W2 1NY, UK; Department of Infectious Diseases, Faculty of Medicine, Imperial College London, London, UK; Imperial College Healthcare NHS Trust, London, UK; National Institute for Health Research Imperial Biomedical Research Centre, Imperial College London, The Bays, Entrance, 2 S Wharf Rd, London W2 1NY, UK; Department of Epidemiology and Biostatistics, School of Public Health, Imperial College London, 90 Wood Ln, London W12 0BZ, UK; National Institute for Health Research Imperial Biomedical Research Centre, Imperial College London, The Bays, Entrance, 2 S Wharf Rd, London W2 1NY, UK; Department of Epidemiology and Biostatistics, School of Public Health, Imperial College London, 90 Wood Ln, London W12 0BZ, UK; National Institute for Health Research Imperial Biomedical Research Centre, Imperial College London, The Bays, Entrance, 2 S Wharf Rd, London W2 1NY, UK; MRC Centre for Environment and Health, School of Public Health, Imperial College London, Praed Street, London W2 1NY, UK; British Heart Foundation Centre for Research Excellence, Imperial College London, South Kensington Campus, London SW7 2AZ, UK; Dementia Research Institute at Imperial College London, 86 Wood Ln, London W12 0BZ, UK; Health Data Research UK, Imperial College London, Exhibition Rd, South Kensington, London SW7 2AZ, UK; Department of Hygiene and Epidemiology, University of Ioannina Medical School, Ioannina, Greece; Department of Epidemiology and Biostatistics, School of Public Health, Imperial College London, 90 Wood Ln, London W12 0BZ, UK; National Institute for Health Research Imperial Biomedical Research Centre, Imperial College London, The Bays, Entrance, 2 S Wharf Rd, London W2 1NY, UK; MRC Centre for Environment and Health, School of Public Health, Imperial College London, Praed Street, London W2 1NY, UK; British Heart Foundation Centre for Research Excellence, Imperial College London, South Kensington Campus, London SW7 2AZ, UK; Dementia Research Institute at Imperial College London, 86 Wood Ln, London W12 0BZ, UK; Health Data Research UK, Imperial College London, Exhibition Rd, South Kensington, London SW7 2AZ, UK; Department of Epidemiology and Biostatistics, School of Public Health, Imperial College London, 90 Wood Ln, London W12 0BZ, UK; MRC Centre for Environment and Health, School of Public Health, Imperial College London, Praed Street, London W2 1NY, UK

**Keywords:** CVD risk prediction, Pooled cohort equations, QRISK3, Biomarkers, Sparse variable selection

## Abstract

**Aims:**

Evaluate sex differences in cardiovascular disease (CVD) risk prediction, including use of (i) optimal sex-specific risk predictors and (ii) sex-specific risk thresholds.

**Methods and results:**

Prospective cohort study using UK Biobank, including 121 724 and 182 632 healthy men and women, respectively, aged 38–73 years at baseline. There were 11 899 (men) and 9110 (women) incident CVD cases (hospitalization or mortality) with a median of 12.1 years of follow-up. We used recalibrated pooled cohort equations (PCEs; 7.5% 10-year risk threshold as per US guidelines), QRISK3 (10% 10-year risk threshold as per UK guidelines), and Cox survival models using sparse sex-specific variable sets (via LASSO stability selection) to predict CVD risk separately in men and women. LASSO stability selection included 12 variables in common between men and women, with 3 additional variables selected for men and 1 for women. *C*-statistics were slightly lower for PCE than QRISK3 and models using stably selected variables, but were similar between men and women: 0.67 (0.66–0.68), 0.70 (0.69–0.71), and 0.71 (0.70–0.72) in men and 0.69 (0.68–0.70), 0.72 (0.71–0.73), and 0.72 (0.71–0.73) in women for PCE, QRISK3, and models using stably selected variables, respectively. At current clinically implemented risk thresholds, test sensitivity was markedly lower in women than men for all models: at 7.5% 10-year risk, sensitivity was 65.1 and 68.2% in men and 24.0 and 33.4% in women for PCE and models using stably selected variables, respectively; at 10% 10-year risk, sensitivity was 53.7 and 52.3% in men and 16.8 and 20.2% in women for QRISK3 and models using stably selected variables, respectively. Specificity was correspondingly higher in women than men. However, the sensitivity in women at 5% 10-year risk threshold increased to 50.1, 58.5, and 55.7% for PCE, QRISK3, and models using stably selected variables, respectively.

**Conclusion:**

Use of sparse sex-specific variables improved CVD risk prediction compared with PCE but not QRISK3. At current risk thresholds, PCE and QRISK3 work less well for women than men, but sensitivity was improved in women using a 5% 10-year risk threshold. Use of sex-specific risk thresholds should be considered in any re-evaluation of CVD risk calculators.

## Graphical Abstract

**Graphical Abstract:**
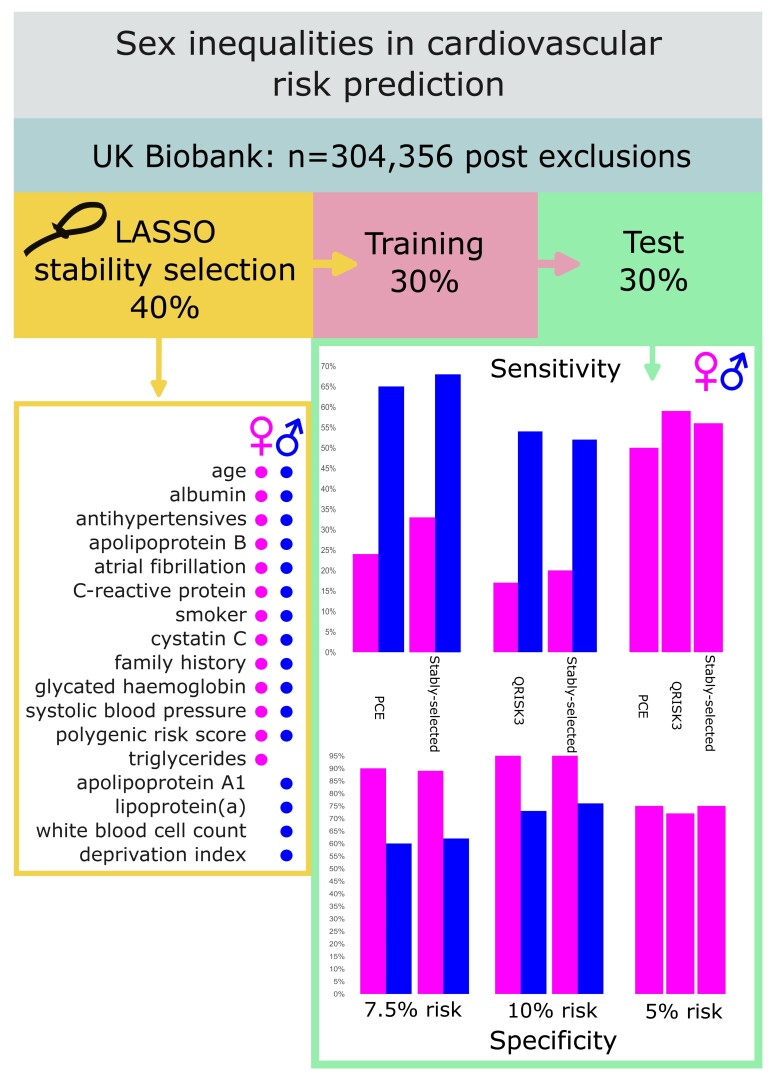

**Time of primary review: 41 days**

**See the editorial comment for this article ‘How far are we from accurate sex-specific risk prediction of cardiovascular disease? One size may not fit all’, by B. Huang *et al*., https://doi.org/10.1093/cvr/cvae135.**

## 1. Introduction

Cardiovascular disease (CVD) is the leading cause of morbidity and mortality worldwide.^1^ Risk stratification via accurate prediction of future CVD risk is key to guiding effective early management and prevention, including lifestyle modifications and lipid-lowering therapeutics. A systematic review of CVD prediction models found that the most commonly included variables were age, smoking, systolic blood pressure, history of diabetes, total cholesterol, and high-density lipoprotein cholesterol.^2^ Alongside ethnicity and history of treated hypertension, these variables are included in the pooled cohort equations (PCEs), which are used in the USA to predict 10-year absolute atherosclerotic CVD risk as a decision aid for recommending lipid-lowering (statin) therapy, with a treatment threshold of 7.5% 10-year absolute risk or greater.^3,4^ In the UK, QRISK3 is used instead and incorporates additional variables, with a 10% 10-year absolute risk of atherosclerotic CVD used as a statin treatment threshold.^5^ However, both the US and UK guidelines note that lower treatment thresholds are likely to be clinically beneficial.^6,7^ Furthermore, there is evidence for sex- and age-specific treatment thresholds, with worse test sensitivity for younger vs. older adults and women vs. men.^8,9^

Models including additional variables have been proposed to predict incident coronary artery disease (CAD), with recent examples combining data from electronic healthcare records with polygenic risk scores (PRSs) for CAD^10^ and blood markers.^11^ Other recent studies have reported that PRS for CAD/CVD, when considered in isolation, yield a modest or non-significant improvement in predictive performance for CVD risk over traditional risk models.^12–16^ It has also been suggested that metabolomic data may be predictive of incident CVD, although their clinical utility in risk prediction remains to be established.^17–20^

Here, we analyse the UK Biobank data set to evaluate sex differences in CVD risk prediction, including use of (i) optimal sex-specific risk predictors and (ii) sex-specific risk thresholds.

## 2. Methods

### 2.1 Study participants

**Figure 1:**
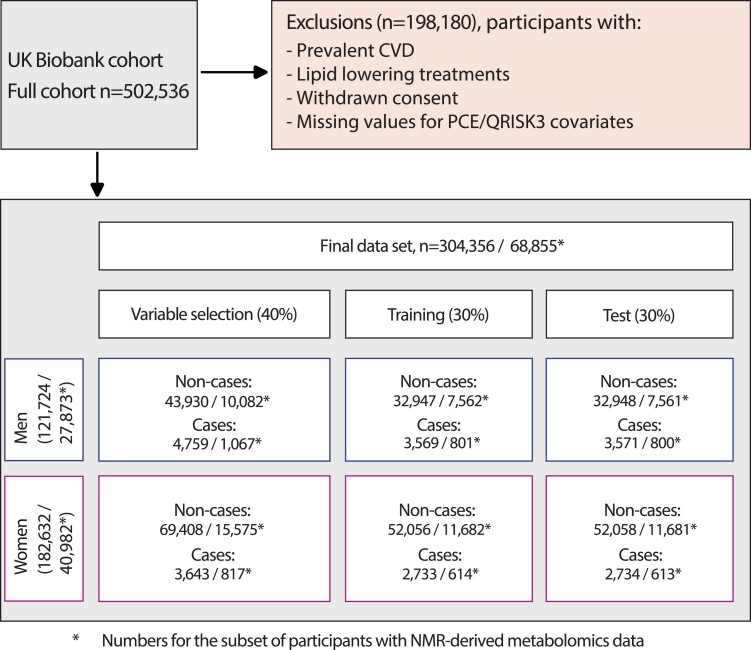
Study design and flowchart. Cases are participants with a CVD event during follow-up including myocardial infarction and its sequelae, angina, non-haemorrhagic stroke, and transient ischaemic attack. Data were randomly split into three sex-stratified, non-overlapping sets: (i) variable selection data set (40%); (ii) training data set (30%), in which Cox models using selected variables were fitted; and (iii) hold-out test data set (30%), in which the predictive accuracy of these models was evaluated and compared with PCEs and QRISK3.

The UK Biobank recruited 502 536 volunteers aged 38–73 years between 2006 and 2010. Demographic and lifestyle factors, medical and surgical histories, standardized clinical measurements, and blood samples were collected at baseline. A panel of laboratory tests was performed on stored serum and red blood cells as well as genotyping.^21^ For the primary analyses, we excluded a total of 198 180 participants: 151 806 with prevalent CVD or missing data for any of the variables included in PCE or QRISK3,^14^ 45 887 on lipid-lowering agents (as PCE and QRISK3 are used to guide the initiation of lipid-lowering therapeutics), and a further 487 who had withdrawn consent, leaving 304 356 participants without prior CVD at baseline for the present analyses (121 724 men and 182 632 women, *Figure 1*). Among these, a subset of 27 873 men and 40 982 women also had data on nuclear magnetic resonance (NMR) metabolic biomarkers measured in baseline plasma samples. The study complies with the Declaration of Helsinki.

### 2.2 CVD definition

CVD was defined as myocardial infarction and its sequelae, angina, non-haemorrhagic stroke, and transient ischaemic attack.^14^ Cases (i.e. people who had a cardiovascular event during follow-up) were identified using linkage to hospital admissions, operation/procedure codes, and death registrations, and prevalent cases were further defined via nurse-administered questionnaire at baseline (see Supplementary material online, *Table S1*). Participants who did not have a recorded cardiovascular event during follow-up are defined here as non-cases, with censoring by availability of hospital admission and mortality data (7 April 2021).

### 2.3 Study variables

Variables included in PCE^3,4^ are age, ethnicity (White, Black, and Other), smoking (never, former, and current), diabetes (prevalent self-reported or from hospital records), total and high-density lipoprotein cholesterol, systolic blood pressure (mean of two measurements), and use of antihypertensive medication. QRISK3^5^ includes additional variables: standard deviation of systolic blood pressure, body mass index, family history of CAD, area-level deprivation score (Townsend), medication use including oral steroids and atypical antipsychotics, and self-reported prevalent conditions including chronic kidney disease stages 3–5, atrial fibrillation, migraine, rheumatoid arthritis, systemic lupus erythematosus, severe mental illness, and erectile dysfunction in men. In addition to the above variables, we considered for variable selection 26 further baseline serum biochemistry measurements (excluding oestradiol and rheumatoid factor that were missing in more than 80% of participants)^22,23^; 23 baseline haematology measurements including full blood count and white blood cell differential^24^; a PRS for CVD developed using lassosum,^25^ as previously described^14^; and NMR-derived metabolic variables (available in ∼120 000 randomly sampled participants from the whole UK Biobank cohort). The NMR-derived metabolomic profile includes estimated blood levels of (*N* = 168) annotated molecules including lipoprotein lipids, fatty acids, and fatty acid compositions, as well as some low-molecular-weight metabolites including amino acids, ketone bodies, and glycolysis metabolites.^26^

### 2.4 Statistical analyses

We randomly split the data into three sex-stratified and non-overlapping sets, constraining the ratio of CVD cases to non-cases to be equal in all three data splits (*Figure 1*): (i) a variable selection data set (40%); (ii) a training data set (30%), in which PCE and QRISK3 were calculated/recalibrated (see Supplementary material online, *Methods* and *Figure S1*) and unpenalized Cox models were fit using stably selected variables; and (iii) a hold-out test data set (30%), comparing the predictive accuracy of recalibrated PCE and QRISK3 with the models using stably selected variables. The Cox models used follow-up time as the underlying time variable with CVD event as outcome. In the subset of participants with NMR data, we compared variable selection and model performance excluding and including metabolomic data. After filtering for highly correlated variables and overlap with directly measured blood markers, 18 metabolomic variables were included in our analyses (see Supplementary material online, *Methods* and *Figure S2*) and were available in 68 855 of the 304 356 participants included in our study (*Figure 1*). For biochemical and haematological variables, there was up to 20% missingness with similar proportions for CVD cases and non-cases (see Supplementary material online, *Table S2*). Missing values were imputed using multiple imputation with predictive mean matching over five iterations of chained random forests.^27^ Skewed variables were log-transformed prior to analyses.

#### 2.4.1 Variable selection

For variable selection, we used LASSO penalized regression in a stability selection framework^28,29^ to identify reproducible, parsimonious sets of variables that jointly contribute to CVD risk prediction. Briefly, we fit LASSO Cox models on (*N* = 1000) 50% independent subsamples of the variable selection data set and estimated, across subsamples, the per-variable selection proportion as a proxy for the variable importance. Model calibration was achieved by jointly identifying (i) the penalty parameter *λ* (controlling the sparsity of the LASSO model) and (ii) the threshold in selection proportion *π* (controlling the stability of the model, conditional on the penalty) above which a feature was considered as stably selected. These parameters were obtained by maximizing a likelihood-based stability score using the *sharp* package in R.^29^ We also performed sensitivity analyses assessing the reliability of the LASSO stability selection, using 100 subsampled variable selection data sets (see Supplementary material online, *Methods*).

#### 2.4.2 Predictive performance

We calculated predictive accuracy (*C*-statistics) as well as sensitivity and specificity at relevant risk thresholds for 10-year risk (7.5% threshold for PCE, 10% threshold for QRISK3, and both 7.5 and 10% thresholds for models using stably selected variables). We used logistic regression models to perform receiver operating characteristic (ROC) analyses, reporting the mean and 95% confidence intervals of the area under the ROC curve (AUC). We also used a nested approach where log hazards from PCE and QRISK3, respectively, were forced into the LASSO stability selection models in place of their constituent variables. In addition, we calculated sensitivity and specificity at 5% 10-year risk threshold in women across all models.

Statistical analyses were performed using R version 4.2.2.^30^

## 3. Results

Mean age at baseline in men was 54.4 years in non-cases and 58.9 years in cases and 55.2 and 60.0 years, respectively, in women. A total of 11 899 men and 9110 women were diagnosed with CVD during the period of follow-up (median 12.1 years). Descriptive statistics, stratified by sex and case status, are shown in Supplementary material online, *Table S3*. Corresponding descriptive statistics for the subset with metabolomic data are reported in Supplementary material online, *Table S4*.

**Figure 2:**
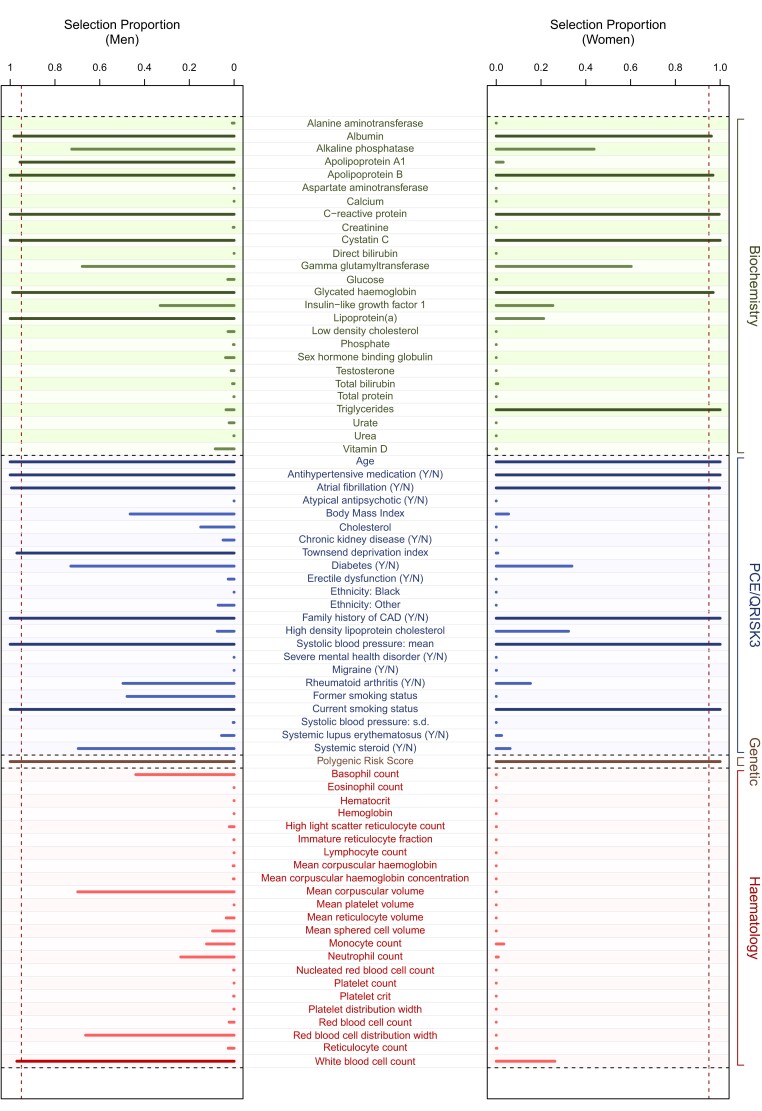
Stability selection LASSO. Selection proportions from LASSO stability selection calculated from (*N* = 1000) subsamples in (*N* = 48 689) men (*left panel*) and (*N* = 73 051) women (*right panel*). Explanatory variables considered include those contributing to PCE and QRISK3 scores (blue), genetic (brown), biochemical (green), and haematological (red) variables. Darker colours indicate stably selected variables (16 and 13 in men and women, respectively) as defined by variables with selection proportion above the calibrated threshold in selection proportion (vertical dark red dashed line).

Our stability selection model consistently selected 12 variables in both men and women (*Figure 2*): age, albumin, antihypertensive medication, apolipoprotein B, atrial fibrillation, C-reactive protein, current smoker, cystatin C, family history of CAD, glycated haemoglobin, systolic blood pressure, and a PRS for CVD. In addition, apolipoprotein A1, lipoprotein(a), white blood cell count, and deprivation index were selected in men only and triglycerides in women only (see Supplementary material online, *Table S5*). Including variables beyond those stably selected did not substantially improve model performance (see Supplementary material online, *Figure S3*).

**Figure 3:**
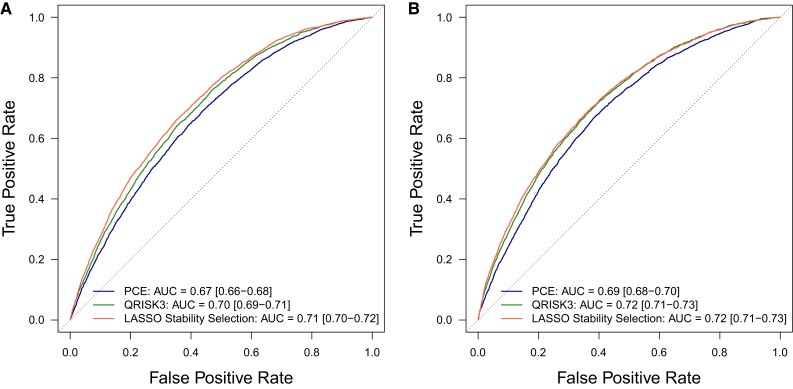
CVD prediction in test data. ROC curves for logistic models predicting 10-year incident CVD in (*N* = 36 519) men (*A*) and (*N* = 54 792) women (*B*) in test data, where models use either recalibrated PCE (blue line), recalibrated QRISK3 (green line), or sex-specific stably selected variables (red line) in test data. We report the mean and 95% confidence intervals for the AUC.

ROC analyses with logistic models for incident CVD in test data showed improvement in predictive accuracy when using stably selected variables vs. recalibrated PCE but not for QRISK3: in men, AUCs were 0.67 (0.66–0.68) for PCE and 0.70 (0.69–0.71) for QRISK3 vs. 0.71 (0.70–0.72) for models using stably selected variables; in women, they were 0.69 (0.68–0.70) for PCE and 0.72 (0.71–0.73) for QRISK3 vs. 0.72 (0.71–0.73) for stably selected variables (*Figure 3*).

*Table 1* shows 10-year risk prediction reclassification, sensitivity, and specificity for LASSO stability selection variables vs. PCE (7.5% risk threshold) and QRISK3 (10% risk threshold). Test sensitivity was markedly lower in women than men for all models: at 7.5% 10-year risk, sensitivity was 65.1 and 68.2% in men and 24.0 and 33.4% in women for PCE and models using stably selected variables, respectively; at 10% 10-year risk, sensitivity was 53.7 and 52.3% in men and 16.8 and 20.2% in women for QRISK3 and stably selected variables, respectively. Specificity was correspondingly higher in women than men. However, the sensitivity in women at 5% 10-year risk threshold increased to 50.1, 58.5, and 55.7% for PCE, QRISK3, and stably selected variables, respectively (*Table 2*).

**Table 1:** Reclassification of CVD cases and non-cases in test data comparing Cox survival models using LASSO stability–selected variables with validated risk prediction algorithms: (A) at 7.5% 10-year risk threshold for PCE and (B) at 10% 10-year risk threshold for QRISK3; sensitivity and specificity are shown at the relevant risk thresholds

A PCE vs. LASSO				
Men				
PCE Predicted 10-year risk (%)		LASSO stability selection		
		Predicted 10-year risk (%)		Reclassified (%)
		<7.5	≥7.5	
Cases	<7.5	808	439	35.2
	≥7.5	329	1995	14.2
Non-cases	<7.5	16 886	2851	14.4
	≥7.5	3610	9601	27.3

	LASSO%	PCE%
Sensitivity (at 7.5% 10-year risk)	68.2	65.1
Specificity (at 7.5% 10-year risk)	62.2	59.9

Women				
PCE Predicted 10-year risk (%)		LASSO stability selection		
		Predicted 10-year risk (%)		Reclassified (%)
		<7.5	≥7.5	
Cases	<7.5	1631	448	21.5
	≥7.5	189	466	28.9
Non-cases	<7.5	43 785	3149	6.7
	≥7.5	2430	2694	47.4

	LASSO%	PCE%
Sensitivity (at 7.5% 10-year risk)	33.4	24.0
Specificity (at 7.5% 10-year risk)	88.8	90.2

B QRISK3 vs. LASSO				
Men				
QRISK3 Predicted 10-year risk (%)		LASSO stability selection		
		Predicted 10-year risk (%)		Reclassified (%)
		<10	≥10	
Cases	<10	1391	264	16.0
	≥10	312	1604	16.3
Non-cases	<10	22 431	1499	6.3
	≥10	2483	6535	27.5

	LASSO%	QRISK3%
Sensitivity (at 10% 10-year risk)	52.3	53.7
Specificity (at 10% 10-year risk)	75.6	72.6

Women				
QRISK3 Predicted 10-year risk (%)		LASSO stability selection		
		Predicted 10-year risk (%)		Reclassified (%)
		<10	≥10	
Cases	<10	2058	218	9.6
	≥10	124	334	27.1
Non-cases	<10	48 269	1358	2.7
	≥10	984	1447	40.5

	LASSO%	QRISK3%
Sensitivity (at 10% 10-year risk)	20.2	16.8
Specificity (at 10% 10-year risk)	94.6	95.3

**Table 2:** Reclassification of CVD cases and non-cases in test data among women using 5% 10-year risk thresholds: (A) recalibrated PCEs and (B) QRISK3 compared with Cox survival models using LASSO stability selected variables; (C) shows sensitivity and specificity for each model at 5% 10-year risk threshold

A PCE vs. LASSO				
PCE Predicted 10-year risk (%)		LASSO stability selection		
		Predicted 10-year risk (%)		Reclassified (%)
		<5	≥5	
Cases	<5	962	402	29.5
	≥5	249	1121	18.2
Non-cases	<5	34 755	4306	11.0
	≥5	4439	8558	34.2

B QRISK3 vs. LASSO				
QRISK3 Predicted 10-year risk (%)		LASSO stability selection		
		Predicted 10-year risk (%)		Reclassified (%)
		<5	≥5	
Cases	<5	959	175	15.4
	≥5	252	1348	15.8
Non-cases	<5	35 380	2285	6.1
	≥5	3814	10 579	26.5

C Sensitivity and specificity at 5% 10-year risk threshold			
	LASSO%	QRISK3%	PCE%
Sensitivity (at 5% 10-year risk)	55.7	58.5	50.1
Specificity (at 5% 10-year risk)	75.3	72.4	75.0

In sensitivity analyses where PCE or QRISK3 log hazards were included in lieu of the constituent variables, stably selected variables differed slightly from the main analyses (see Supplementary material online, *Figure S4*). This did not affect model performances, with similar *C*-statistics, sensitivity, and specificity (see Supplementary material online, *Table S6*).

Among the subset of (*N* = 68 855) participants with available metabolomic data, glycoprotein acetyls was selected in women only, in preference to C-reactive protein (see Supplementary material online, *Figure S5*), with no improvement in predictive performance (see Supplementary material online, *Figures S6* and *S7*). Assessment of the reliability of LASSO stability selection showed similar variable sets across 100 subsampled iterations (see Supplementary material online, *Methods* and *Figure S8*).

## 4. Discussion

In this large population-based cohort, use of sex-specific stably selected variables improved predictive performance for CVD beyond PCE but not QRISK3, although QRISK3 was also developed selecting from an extensive set of risk predictors.^5^ Among the variables selected in both men and women, some are already included in PCE and QRISK3, while others used in these risk calculators were not selected (diabetes status, ethnicity, high-density lipoprotein, and total cholesterol). At the current clinical risk thresholds, sensitivity was much lower in women (with higher specificity) than in men for both PCE and QRISK3. A higher proportion of incident CVD cases might therefore go untreated in women than men using a common risk threshold for both sexes, as is current practice.

Our results concerning test sensitivity by sex are consistent with previous findings for PCE. In an analysis of PCE among 3685 participants in the Framingham Offspring Study, sensitivity was lower in women than men at the clinically used 7.5% 10-year risk threshold, except at the oldest ages; the authors suggest using a 5% risk threshold at younger ages (40–55 years).^8^ In 1685 patients of the YOUNG-MI registry, who had a myocardial infarction aged 50 years or below, sensitivity of PCE in women was around half that in men at the 7.5% risk threshold. However, sensitivity in women at the 5% risk threshold was similar to that in men at the 7.5% threshold.^9^ Together with our own findings, these results suggest that sex-specific risk thresholds should be considered for clinical implementation to avoid sex inequality in CVD risk prediction.

Sex-specific differences in CVD risk prediction are not well understood. They may reflect underlying physiological differences, including the impact of sex hormones, vascular remodelling, lipid metabolism, and endothelial function.^31,32^ In our study, among lipids, triglycerides^33^ were selected in women only and lipoprotein(a)^34,35^ and apolipoprotein A1^36–38^ in men only. Apolipoprotein B was selected in both men and women, replacing more standard lipid measures currently included in PCE and QRISK3, consistent with it being a better risk predictor of incident CVD.^39,40^ In keeping with this, both the European Society of Cardiology^41^ and the 2019 American College of Cardiology/American Heart Association guidelines on primary prevention of CVD^42^ have highlighted the utility of apolipoprotein B to improve risk stratification.

Systemic inflammation is an important component of CVD risk, and some of the selected variables reflect this: while white blood cell count was selected in men only, serum albumin,^43^ C-reactive protein^44^ (acute phase reactants), and cystatin C (a sensitive marker of renal function^45,46^) were selected in both men and women. Among NMR metabolomic variables, glycoprotein acetyls^47–49^ were selected in women only in preference to C-reactive protein, but this did not improve predictive accuracy. In addition, glycated haemoglobin, a biomarker used in the diagnosis and monitoring of diabetes and non-diabetic hyperglycaemia^50^ (both pro-inflammatory states),^51^ was stably selected in preference to diabetes status in both men and women, in keeping with it being a continuous and therefore more informative variable. Given that glycated haemoglobin is increasingly recorded in electronic health records and offers a superior predictor of CVD risk, a strong case can be made for its inclusion in CVD risk calculators.

Use of PRS in CVD risk prediction remains controversial^14,52^; here, it was stably selected in both men and women but made only modest contribution to predictive accuracy, in keeping with previous analyses of UK Biobank and other data.^14,15^ Family history of CAD, which may reflect common lifestyle and socio-economic factors as well as genetic risk,^34^ was also stably selected alongside PRS, indicating that they both jointly and independently contribute to CVD risk.

### 4.1 Limitations

We only included participants aged 38–73 years at baseline who were mostly of European ancestry; the participants were on average healthier, were less deprived, and have lower mortality than the general population and therefore may not be fully representative.^53^ While PCE was developed in US cohorts, the present study uses a UK-based cohort; we performed model recalibration to correct for population differences^54^ and included the standard risk prediction tool (QRISK3) used in the UK. Other potentially important predictors including coronary artery calcium were not measured, and their inclusion may further improve risk prediction or potentially compete with variables selected in our models.^55^ The UK Biobank does not have complete prescription data during follow-up, so it is likely that some participants’ CVD risk would have been modified from baseline through clinical management. Cost–benefit and decision analyses would be needed before implementing either sex-specific risk thresholds or an enhanced predictive score. Variable selection, training, and test data were drawn from the same population; external validation in different cohorts and settings would help to generalize our findings to other populations.

### 4.2. Conclusions

Use of sparse sex-specific variables improved CVD risk prediction compared with PCE but not QRISK3. At current risk thresholds, PCE and QRISK3 work less well for women than men, but sensitivity was improved in women using a 5% 10-year risk threshold. Use of sex-specific risk thresholds should be considered in any re-evaluation of CVD risk calculators.

**Translational perspective**

Cardiovascular disease risk prediction is an important component of clinical risk management and disease prevention. We find that at risk prediction thresholds used by currently applied risk prediction algorithms (pooled cohort equation 7.5% 10-year risk threshold in the USA and QRISK3 10% risk threshold in the UK), sensitivity of these risk prediction tools is markedly lower in women than in men. This sex inequality implies that women are proportionately less likely to receive appropriate clinical management including lipid-lowering therapy. If the risk prediction threshold is lowered to 5% 10-year risk in women, then sensitivity in women is substantially increased.

## Supplementary Material

cvae123_Supplementary_Data

## Data Availability

This study was conducted using the UK Biobank resource under application number 69328 granting access to the corresponding UK Biobank genetic and phenotype data. The UK Biobank received ethical approval from the North West Multi-centre Research Ethics Committee (REC reference: 11/NW/0382) to obtain and disseminate data and samples from the participants (http://www.ukbiobank.ac.uk/ethics/).
