# Supplementary material for: Sex inequalities in cardiovascular risk prediction
Source: Cardiovasc Res. 2024 Jun 4;120(11):1327–35. doi: 10.1093/cvr/cvae123 (PMC11416056; doi:10.1093/cvr/cvae123)
Supplement: cvae123_Supplementary_Data [file cvae123_supplementary_data.docx]

**SUPPLEMENTARY Material**

Sex inequalities in cardiovascular risk prediction

Elliott J, Bodinier B, Whitaker M, et al.

Supplementary Tables 1-6

Supplementary Figures 1-8

Supplementary Methods

**Supplementary Table 1.** Case definition for cardiovascular disease

| **ICD-10** | **ICD-9** | **OPCS-4** | **Biobank field:**  **20002** | **Biobank field:**  **20004** | **Biobank field:**  **6150** |
| --- | --- | --- | --- | --- | --- |
| G45 | 410 | K40 | 1074: Angina | 1070: Coronary angioplasty | 1: Heart attack |
| I20 | 411 | K41 | 1075: Heart attack/myocardial infarction | 1071: Other arterial surgery/revascularization  procedures | 2: Angina |
| I21 | 412 | K42 | 1082: Transient ischemic attack | 1095: Coronary artery bypass grafts | 3: Stroke |
| I22 | 413 | K43 | 1583: Ischemic stroke | 1105: Carotid artery surgery/endarterectomy |  |
| I23 | 414 | K44 |  | 1109: Carotid artery angioplasty +/- stent |  |
| I24 | 434 | K45 |  | 1514: Coronary angiogram |  |
| I25 | 436 | K46 |  |  |  |
| I63 |  | K47.1 |  |  |  |
| I64 |  | K49 |  |  |  |
|  |  | K50 |  |  |  |
|  |  | K75 |  |  |  |

**Supplementary Table 2.** Missingness (%) by variable prior to imputation, stratified by case status and sex.

|  | ***Men*** | | ***Women*** | |
| --- | --- | --- | --- | --- |
| **Variable** | **Cases** | **Non-cases** | **Cases** | **Non-cases** |
| Alanine aminotransferase (U/L) | 0.09 | 0.09 | 0.01 | 0.03 |
| Albumin (g/L) | 0.04 | 0.04 | 0.04 | 0.04 |
| Alkaline phosphatase (U/L) | 0.02 | 0.02 | 0.01 | 0.01 |
| Apolipoprotein A1 (g/L) | 0.29 | 0.18 | 1.00 | 0.94 |
| Apolipoprotein B (g/L) | 0.73 | 0.66 | 0.53 | 0.36 |
| Aspartate aminotransferase (U/L) | 0.45 | 0.41 | 0.41 | 0.40 |
| Basophil count (e9 cells/L) | 2.40 | 2.49 | 2.93 | 2.81 |
| C-reactive protein (mg/L) | 0.33 | 0.25 | 0.26 | 0.18 |
| Calcium (mmol/L) | 0.07 | 0.07 | 0.08 | 0.07 |
| Creatinine (µmol/L) | 0.10 | 0.07 | 0.07 | 0.07 |
| Cystatin C (mg/L) | 0.06 | 0.07 | 0.05 | 0.05 |
| Direct bilirubin (µmol/L) | 8.57 | 7.21 | 27.67 | 22.26 |
| Eosinophil count (e9 cells/L) | 2.40 | 2.49 | 2.93 | 2.81 |
| Gamma glutamyltransferase (U/L) | 0.12 | 0.07 | 0.07 | 0.06 |
| Glucose (mmol/L) | 0.12 | 0.13 | 0.08 | 0.11 |
| Glycated hemoglobin (mmol/mol) | 4.48 | 4.85 | 4.96 | 4.78 |
| Hematocrit (%) | 2.27 | 2.33 | 2.78 | 2.63 |
| Hemoglobin concentration (g/dL) | 2.27 | 2.33 | 2.78 | 2.63 |
| High light scatter reticulocyte count (e12 cells/L) | 3.46 | 3.88 | 4.22 | 4.28 |
| Immature reticulocyte fraction | 3.46 | 3.88 | 4.22 | 4.28 |
| Insulin-like growth factor 1 (nmol/L) | 0.57 | 0.59 | 0.83 | 0.63 |
| Lipoprotein(a) (g/L) | 19.50 | 19.19 | 20.02 | 18.98 |
| Low-density lipoprotein cholesterol (mmol/L) | 0.18 | 0.17 | 0.15 | 0.16 |
| Lymphocyte count (e9 cells/L) | 2.40 | 2.49 | 2.93 | 2.81 |
| Mean corpuscular hemoglobin (pg) | 2.27 | 2.33 | 2.78 | 2.63 |
| Mean corpuscular hemoglobin concentration (g/dL) | 2.27 | 2.33 | 2.78 | 2.63 |
| Mean corpuscular volume (fL) | 2.27 | 2.33 | 2.78 | 2.63 |
| Mean platelet volume (fL) | 2.27 | 2.33 | 2.78 | 2.63 |
| Mean reticulocyte volume (fL) | 3.46 | 3.88 | 4.22 | 4.28 |
| Mean sphered cell volume (fL) | 3.46 | 3.88 | 4.22 | 4.28 |
| Monocyte count (e9 cells/L) | 2.40 | 2.49 | 2.93 | 2.81 |
| Neutrophil count (e9 cells/L) | 2.40 | 2.49 | 2.93 | 2.81 |
| Nucleated red blood cell count (e9 cells/L) | 2.40 | 2.50 | 2.93 | 2.81 |
| Phosphate (mmol/L) | 0.29 | 0.23 | 0.25 | 0.23 |
| Platelet count (e9 cells/L) | 2.27 | 2.33 | 2.78 | 2.63 |
| Platelet crit (%) | 2.27 | 2.33 | 2.78 | 2.63 |
| Platelet distribution width (%) | 2.27 | 2.33 | 2.78 | 2.63 |
| Red blood cell count (e12 cells/L) | 2.27 | 2.33 | 2.78 | 2.63 |
| Red blood cell distribution width (%) | 2.27 | 2.33 | 2.78 | 2.63 |
| Reticulocyte count (e9 cells/L) | 3.46 | 3.88 | 4.22 | 4.28 |
| Sex hormone binding globulin (nmol/L) | 0.69 | 0.86 | 1.20 | 1.23 |
| Testosterone (nmol/L) | 1.03 | 0.94 | 19.78 | 15.09 |
| Total bilirubin (µmol/L) | 0.50 | 0.43 | 0.44 | 0.44 |
| Total protein (g/L) | 0.18 | 0.16 | 0.15 | 0.13 |
| Triglycerides (mmol/L) | 0.11 | 0.08 | 0.04 | 0.07 |
| Urate (µmol/L) | 0.13 | 0.11 | 0.10 | 0.13 |
| Urea (mmol/L) | 0.13 | 0.09 | 0.07 | 0.09 |
| Vitamin D (µg) | 5.19 | 3.64 | 9.69 | 6.20 |
| White blood cell count (e9 cells/L) | 2.27 | 2.33 | 2.78 | 2.63 |

**Supplementary Table 3.** Descriptive statistics of data with imputation, stratified by case status for (A) men and (B) women. For continuous variables, mean values (s.d.) shown and for binary variables, prevalence of “yes” (%) is shown.

**(A)**

| **Variable** | **Non-cases** | **Cases** |
| --- | --- | --- |
| *N* | 109,825 | 11,899 |
|  |  | |
|  | **Mean (s.d.)** | |
| Age (years) | 54.40 (8.30) | 58.88 (7.40) |
| Body mass index (kg/m2) | 27.30 (3.98) | 27.98 (4.20) |
| Systolic blood pressure: mean (mmHg) | 139.58 (17.04) | 145.78 (18.30) |
| Systolic blood pressure: s.d. (mmHg) | 5.13 (4.25) | 5.49 (4.50) |
| Townsend deprivation index | -1.37 (3.07) | -1.23 (3.17) |
| Alanine aminotransferase (U/L) | 27.14 (15.48) | 26.84 (15.64) |
| Albumin (g/L) | 45.65 (2.57) | 45.05 (2.59) |
| Alkaline phosphatase (U/L) | 81.18 (23.58) | 84.88 (28.54) |
| Apolipoprotein A1 (g/L) | 1.44 (0.23) | 1.41 (0.23) |
| Apolipoprotein B (g/L) | 1.07 (0.23) | 1.12 (0.23) |
| Aspartate aminotransferase (U/L) | 27.93 (11.42) | 28.08 (11.77) |
| Calcium (mmol/L) | 2.37 (0.09) | 2.37 (0.09) |
| Cholesterol (mmol/L) | 222.27 (38.91) | 228.67 (40.18) |
| C-reactive protein (mg/L) | 2.31 (4.13) | 3.07 (4.91) |
| Creatinine (µmol/L) | 80.70 (13.56) | 81.75 (20.84) |
| Cystatin C (mg/L) | 0.92 (0.14) | 0.97 (0.18) |
| Direct bilirubin (µmol/L) | 1.93 (0.89) | 1.88 (0.90) |
| Gamma glutamyltransferase (U/L) | 43.25 (45.33) | 47.41 (50.93) |
| Glucose (mmol/L) | 5.00 (0.99) | 5.10 (1.25) |
| Glycated hemoglobin (mmol/mol) | 34.95 (5.38) | 36.23 (6.71) |
| High-density lipoprotein cholesterol (mmol/L) | 50.41 (11.97) | 49.00 (11.94) |
| Insulin-like growth factor 1 (nmol/L) | 22.33 (5.41) | 21.48 (5.50) |
| Lipoprotein(a) (g/L) | 42.36 (47.75) | 46.76 (49.19) |
| Low density cholesterol (mmol/L) | 3.70 (0.77) | 3.84 (0.80) |
| Phosphate (mmol/L) | 1.11 (0.16) | 1.11 (0.16) |
| Sex hormone binding globulin (nmol/L) | 39.43 (16.62) | 41.57 (17.56) |
| Testosterone (nmol/L) | 12.34 (3.70) | 12.18 (3.78) |
| Total bilirubin (µmol/L) | 10.34 (4.90) | 10.03 (4.60) |
| Total protein (g/L) | 72.66 (4.04) | 72.50 (4.16) |
| Triglycerides (mmol/L) | 1.94 (1.14) | 2.08 (1.19) |
| Urate (µmol/L) | 350.83 (68.56) | 358.73 (72.64) |
| Urea (mmol/L) | 5.48 (1.26) | 5.63 (1.48) |
| Vitamin D (µg) | 48.04 (20.99) | 47.60 (20.68) |
| Basophil count (e9 cells/L) | 0.03 (0.04) | 0.04 (0.05) |
| Eosinophil count (e9 cells/L) | 0.18 (0.14) | 0.19 (0.15) |
| Hematocrit (%) | 43.47 (2.87) | 43.61 (3.08) |
| Hemoglobin concentration (g/dL) | 15.06 (0.97) | 15.10 (1.04) |
| High light scatter reticulocyte count (e12 cells/L) | 0.02 (0.01) | 0.02 (0.01) |
| Immature reticulocyte fraction | 0.28 (0.06) | 0.29 (0.06) |
| Lymphocyte count (e9 cells/L) | 1.88 (1.29) | 1.96 (2.06) |
| Mean corpuscular hemoglobin (pg) | 31.64 (1.79) | 31.78 (1.83) |
| Mean corpuscular hemoglobin concentration (g/dL) | 34.67 (1.03) | 34.64 (1.03) |
| Mean corpuscular volume (fL) | 91.28 (4.37) | 91.75 (4.54) |
| Mean platelet volume (fL) | 9.27 (1.06) | 9.25 (1.06) |
| Mean reticulocyte volume (fL) | 106.09 (7.65) | 106.84 (7.82) |
| Mean sphered cell volume (fL) | 82.72 (5.26) | 83.23 (5.44) |
| Monocyte count (e9 cells/L) | 0.50 (0.21) | 0.53 (0.25) |
| Neutrophil count (e9 cells/L) | 4.13 (1.39) | 4.40 (1.47) |
| Nucleated red blood cell count (e9 cells/L) | 0.02 (0.03) | 0.02 (0.02) |
| Platelet count (e9 cells/L) | 239.43 (54.72) | 239.64 (58.49) |
| Platelet crit (%) | 0.22 (0.04) | 0.22 (0.05) |
| Platelet distribution width (%) | 16.55 (0.52) | 16.58 (0.53) |
| Red blood cell count (e12 cells/L) | 4.77 (0.37) | 4.76 (0.39) |
| Red blood cell distribution width (%) | 13.39 (0.83) | 13.52 (0.89) |
| Reticulocyte count (e9 cells/L) | 0.06 (0.04) | 0.07 (0.04) |
| White blood cell count (e9 cells/L) | 6.73 (2.13) | 7.12 (2.76) |
| Polygenic risk score | -0.03 (0.78) | 0.15 (0.79) |
|  | **Prevalence of “yes” (%)** | |
| Antihypertensive medication | 12,546 (11.42) | 2,501 (21.02) |
| Atrial fibrillation | 888 (0.81) | 250 (2.10) |
| Atypical antipsychotic | 290 (0.26) | 29 (0.24) |
| Chronic kidney disease (stages 3-5) | 45 (0.04) | 15 (0.13) |
| Diabetes | 2,347 (2.14) | 540 (4.54) |
| Erectile dysfunction | 463 (0.42) | 89 (0.75) |
| Ethnicity: Black | 2,014 (1.83) | 119 (1.00) |
| Ethnicity: Other | 4,251 (3.87) | 428 (3.60) |
| Family history of coronary artery disease | 38,491 (35.05) | 5,241 (44.05) |
| Migraine | 1,733 (1.58) | 179 (1.50) |
| Rheumatoid arthritis | 627 (0.57) | 148 (1.24) |
| Severe mental health disorder | 503 (0.46) | 61 (0.51) |
| Smoker: current | 12,473 (11.36) | 1,809 (15.20) |
| Smoker: former | 38,870 (35.39) | 4,857 (40.82) |
| Systemic lupus erythematosus | 29 (0.03) | 8 (0.07) |
| Systemic steroid | 690 (0.63) | 149 (1.25) |

**(B)**

| **Variable** | **Non-cases** | **Cases** |
| --- | --- | --- |
| *N* | 173,522 | 9,110 |
|  |  |  |
|  | **Mean (s.d.)** | |
| Age (years) | 55.15 (7.98) | 60.00 (6.89) |
| Body mass index (kg/m2) | 26.59 (4.92) | 27.86 (5.33) |
| Systolic blood pressure: mean (mmHg) | 133.85 (18.96) | 142.22 (20.12) |
| Systolic blood pressure: s.d. (mmHg) | 5.40 (4.45) | 5.86 (4.84) |
| Townsend deprivation index | -1.46 (2.97) | -1.22 (3.10) |
| Alanine aminotransferase (U/L) | 19.52 (11.86) | 20.97 (12.62) |
| Albumin (g/L) | 44.98 (2.57) | 44.51 (2.63) |
| Alkaline phosphatase (U/L) | 83.07 (26.76) | 91.26 (29.32) |
| Apolipoprotein A1 (g/L) | 1.65 (0.27) | 1.61 (0.28) |
| Apolipoprotein B (g/L) | 1.05 (0.23) | 1.13 (0.24) |
| Aspartate aminotransferase (U/L) | 24.04 (9.30) | 25.15 (10.22) |
| Calcium (mmol/L) | 2.38 (0.10) | 2.39 (0.10) |
| Cholesterol (mmol/L) | 231.02 (41.72) | 242.86 (42.61) |
| C-reactive protein (mg/L) | 2.56 (4.17) | 3.55 (5.07) |
| Creatinine (µmol/L) | 63.77 (10.76) | 64.81 (18.65) |
| Cystatin C (mg/L) | 0.86 (0.14) | 0.93 (0.20) |
| Direct bilirubin (µmol/L) | 1.53 (0.65) | 1.45 (0.62) |
| Gamma glutamyltransferase (U/L) | 28.65 (31.78) | 34.49 (38.78) |
| Glucose (mmol/L) | 4.97 (0.81) | 5.11 (1.10) |
| Glycated hemoglobin (mmol/mol) | 34.94 (4.56) | 36.49 (5.89) |
| High-density lipoprotein cholesterol (mmol/L) | 62.44 (14.45) | 59.71 (14.39) |
| Insulin-like growth factor 1 (nmol/L) | 21.27 (5.72) | 19.78 (5.68) |
| Lipoprotein(a) (g/L) | 44.74 (49.02) | 48.22 (50.32) |
| Low density cholesterol (mmol/L) | 3.71 (0.84) | 3.98 (0.85) |
| Phosphate (mmol/L) | 1.19 (0.15) | 1.20 (0.15) |
| Sex hormone binding globulin (nmol/L) | 64.41 (31.45) | 60.76 (30.85) |
| Testosterone (nmol/L) | 1.11 (0.62) | 1.14 (0.67) |
| Total bilirubin (µmol/L) | 8.16 (3.70) | 7.80 (3.41) |
| Total protein (g/L) | 72.40 (4.09) | 72.33 (4.22) |
| Triglycerides (mmol/L) | 1.49 (0.82) | 1.78 (0.94) |
| Urate (µmol/L) | 264.68 (62.27) | 282.77 (68.47) |
| Urea (mmol/L) | 5.13 (1.24) | 5.42 (1.41) |
| Vitamin D (µg) | 48.60 (20.79) | 47.10 (20.29) |
| Basophil count (e9 cells/L) | 0.04 (0.05) | 0.04 (0.06) |
| Eosinophil count (e9 cells/L) | 0.16 (0.13) | 0.17 (0.13) |
| Hematocrit (%) | 39.16 (2.79) | 39.55 (2.88) |
| Hemoglobin concentration (g/dL) | 13.47 (0.96) | 13.60 (0.98) |
| High light scatter reticulocyte count (e12 cells/L) | 0.02 (0.01) | 0.02 (0.01) |
| Immature reticulocyte fraction | 0.29 (0.06) | 0.30 (0.06) |
| Lymphocyte count (e9 cells/L) | 1.99 (0.94) | 2.08 (0.76) |
| Mean corpuscular hemoglobin (pg) | 31.29 (1.95) | 31.32 (1.96) |
| Mean corpuscular hemoglobin concentration (g/dL) | 34.41 (1.05) | 34.41 (1.10) |
| Mean corpuscular volume (fL) | 90.90 (4.69) | 90.99 (4.69) |
| Mean platelet volume (fL) | 9.37 (1.09) | 9.33 (1.09) |
| Mean reticulocyte volume (fL) | 105.57 (7.75) | 105.94 (8.08) |
| Mean sphered cell volume (fL) | 83.10 (5.30) | 83.24 (5.55) |
| Monocyte count (e9 cells/L) | 0.43 (0.20) | 0.46 (0.20) |
| Neutrophil count (e9 cells/L) | 4.14 (1.37) | 4.37 (1.51) |
| Nucleated red blood cell count (e9 cells/L) | 0.02 (0.04) | 0.02 (0.02) |
| Platelet count (e9 cells/L) | 266.18 (60.12) | 270.53 (63.78) |
| Platelet crit (%) | 0.25 (0.05) | 0.25 (0.05) |
| Platelet distribution width (%) | 16.42 (0.51) | 16.44 (0.51) |
| Red blood cell count (e12 cells/L) | 4.31 (0.33) | 4.35 (0.35) |
| Red blood cell distribution width (%) | 13.51 (1.06) | 13.61 (1.08) |
| Reticulocyte count (e9 cells/L) | 0.06 (0.03) | 0.06 (0.04) |
| White blood cell count (e9 cells/L) | 6.76 (1.88) | 7.10 (1.91) |
| Polygenic risk score | 0.00 (0.79) | 0.15 (0.80) |
|  | **Prevalence of “yes” (%)** | |
| Antihypertensive medication | 20,247 (11.67) | 2,221 (24.38) |
| Atrial fibrillation | 561 (0.32) | 140 (1.54) |
| Atypical antipsychotic | 339 (0.20) | 26 (0.29) |
| Chronic kidney disease (stages 3-5) | 47 (0.03) | 11 (0.12) |
| Diabetes | 2,165 (1.25) | 281 (3.08) |
| Ethnicity: Black | 3,205 (1.85) | 123 (1.35) |
| Ethnicity: Other | 5,947 (3.43) | 302 (3.32) |
| Family history of CAD | 73,224 (42.20) | 4,895 (53.73) |
| Migraine | 7,701 (4.44) | 446 (4.90) |
| Rheumatoid arthritis | 2,227 (1.28) | 233 (2.56) |
| Severe mental health disorder | 694 (0.40) | 64 (0.70) |
| Smoker: current | 13,447 (7.75) | 1,146 (12.58) |
| Smoker: former | 54,916 (31.65) | 3,160 (34.69) |
| Systemic lupus erythematosus | 314 (0.18) | 45 (0.49) |
| Systemic steroid | 1,306 (0.75) | 156 (1.71) |

**Supplementary Table 4.** Descriptive statistics for the subset with NMR-derived metabolomic data, stratified by case status, for (A) men and (B) women. For continuous variables, mean values (s.d.) shown and for binary variables, prevalence of “yes” (%) is shown.

**(A)**

| **Variable** | **Non-cases** | **Cases** |
| --- | --- | --- |
| *N* | 25,205 | 2,668 |
|  |  | |
|  | **Mean (s.d.)** | |
| Age (years) | 54.36 (8.30) | 58.92 (7.41) |
| Body mass index (kg/m2) | 27.30 (3.97) | 27.99 (4.28) |
| Systolic blood pressure: mean (mmHg) | 139.54 (16.96) | 145.73 (18.40) |
| Systolic blood pressure: s.d. (mmHg) | 5.14 (4.23) | 5.52 (4.56) |
| Townsend deprivation index | -1.40 (3.07) | -1.25 (3.15) |
| Alanine aminotransferase (U/L) | 27.26 (16.03) | 26.35 (15.30) |
| Albumin (g/L) | 45.69 (2.55) | 45.04 (2.59) |
| Alkaline phosphatase (U/L) | 81.02 (23.81) | 83.51 (23.89) |
| Apolipoprotein A1 (g/L) | 1.43 (0.23) | 1.41 (0.23) |
| Apolipoprotein B (g/L) | 1.07 (0.23) | 1.12 (0.23) |
| Aspartate aminotransferase (U/L) | 27.88 (12.06) | 27.63 (10.71) |
| Calcium (mmol/L) | 2.37 (0.09) | 2.37 (0.09) |
| Cholesterol (mmol/L) | 222.15 (38.96) | 227.51 (39.15) |
| C-reactive protein (mg/L) | 2.31 (4.18) | 3.00 (4.92) |
| Creatinine (µmol/L) | 80.85 (13.45) | 81.53 (14.06) |
| Cystatin C (mg/L) | 0.92 (0.13) | 0.97 (0.16) |
| Direct bilirubin (µmol/L) | 1.94 (0.93) | 1.85 (0.76) |
| Gamma glutamyltransferase (U/L) | 42.72 (41.52) | 46.00 (46.09) |
| Glucose (mmol/L) | 5.00 (0.95) | 5.11 (1.32) |
| Glycated hemoglobin (mmol/mol) | 34.87 (5.21) | 36.16 (6.64) |
| High-density lipoprotein cholesterol (mmol/L) | 50.38 (11.89) | 48.95 (11.96) |
| Insulin-like growth factor 1 (nmol/L) | 22.37 (5.37) | 21.44 (5.35) |
| Lipoprotein(a) (g/L) | 42.52 (47.88) | 47.29 (49.46) |
| Low density cholesterol (mmol/L) | 3.70 (0.77) | 3.82 (0.78) |
| Phosphate (mmol/L) | 1.11 (0.16) | 1.11 (0.16) |
| Sex hormone binding globulin (nmol/L) | 39.42 (16.53) | 41.76 (17.54) |
| Testosterone (nmol/L) | 12.36 (3.69) | 12.17 (3.83) |
| Total bilirubin (µmol/L) | 10.37 (4.96) | 9.89 (4.29) |
| Total protein (g/L) | 72.64 (4.03) | 72.44 (4.21) |
| Triglycerides (mmol/L) | 1.92 (1.12) | 2.05 (1.18) |
| Urate (µmol/L) | 350.62 (68.05) | 357.56 (73.32) |
| Urea (mmol/L) | 5.49 (1.26) | 5.65 (1.36) |
| Vitamin D (µg) | 47.92 (20.86) | 48.10 (20.60) |
| Basophil count (e9 cells/L) | 0.03 (0.04) | 0.04 (0.05) |
| Eosinophil count (e9 cells/L) | 0.18 (0.14) | 0.19 (0.15) |
| Hematocrit (%) | 43.45 (2.85) | 43.48 (3.11) |
| Hemoglobin concentration (g/dL) | 15.06 (0.97) | 15.07 (1.05) |
| High light scatter reticulocyte count (e12 cells/L) | 0.02 (0.01) | 0.02 (0.01) |
| Immature reticulocyte fraction | 0.28 (0.06) | 0.29 (0.06) |
| Lymphocyte count (e9 cells/L) | 1.88 (1.31) | 1.94 (1.33) |
| Mean corpuscular hemoglobin (pg) | 31.64 (1.77) | 31.79 (2.02) |
| Mean corpuscular hemoglobin concentration (g/dL) | 34.68 (1.02) | 34.68 (1.14) |
| Mean corpuscular volume (fL) | 91.26 (4.35) | 91.66 (4.69) |
| Mean platelet volume (fL) | 9.27 (1.07) | 9.24 (1.06) |
| Mean reticulocyte volume (fL) | 106.11 (7.57) | 106.81 (8.00) |
| Mean sphered cell volume (fL) | 82.67 (5.16) | 83.14 (5.58) |
| Monocyte count (e9 cells/L) | 0.50 (0.21) | 0.53 (0.24) |
| Neutrophil count (e9 cells/L) | 4.12 (1.40) | 4.41 (1.46) |
| Nucleated red blood cell count (e9 cells/L) | 0.02 (0.04) | 0.02 (0.02) |
| Platelet count (e9 cells/L) | 239.72 (55.03) | 239.16 (56.65) |
| Platelet crit (%) | 0.22 (0.04) | 0.22 (0.05) |
| Platelet distribution width (%) | 16.56 (0.52) | 16.59 (0.51) |
| Red blood cell count (e12 cells/L) | 4.77 (0.37) | 4.75 (0.40) |
| Red blood cell distribution width (%) | 13.38 (0.82) | 13.51 (0.92) |
| Reticulocyte count (e9 cells/L) | 0.06 (0.03) | 0.06 (0.03) |
| White blood cell count (e9 cells/L) | 6.71 (2.11) | 7.12 (2.28) |
| 3-Hydroxybutyrate (mmol/l) | 0.06 (0.06) | 0.06 (0.06) |
| Acetate (mmol/l) | 0.02 (0.01) | 0.02 (0.01) |
| Acetoacetate (mmol/l) | 0.01 (0.01) | 0.01 (0.01) |
| Acetone (mmol/l) | 0.01 (0.01) | 0.01 (0.01) |
| Alanine (mmol/l) | 0.30 (0.07) | 0.30 (0.07) |
| Citrate (mmol/l) | 0.06 (0.01) | 0.06 (0.01) |
| Fatty acids: degree of unsaturation | 1.34 (0.08) | 1.33 (0.08) |
| Glutamine (mmol/l) | 0.54 (0.08) | 0.54 (0.08) |
| Glycine (mmol/l) | 0.14 (0.04) | 0.14 (0.04) |
| Glycoprotein acetyls (mmol/l) | 0.78 (0.11) | 0.81 (0.11) |
| Histidine (mmol/l) | 0.07 (0.01) | 0.06 (0.01) |
| Lactate (mmol/l) | 3.87 (1.06) | 3.84 (1.05) |
| Omega-3 fatty acids (mmol/l) | 0.49 (0.21) | 0.49 (0.21) |
| Phenylalanine (mmol/l) | 0.05 (0.01) | 0.05 (0.01) |
| Pyruvate (mmol/l) | 0.07 (0.03) | 0.08 (0.03) |
| Total branched-chain amino acids (mmol/l) | 0.37 (0.08) | 0.38 (0.08) |
| Total cholines (mmol/l) | 2.43 (0.36) | 2.45 (0.36) |
| Tyrosine (mmol/l) | 0.06 (0.01) | 0.06 (0.01) |
| Polygenic risk score | -0.03 (0.78) | 0.18 (0.81) |
|  | **Prevalence of “yes” (%)** | |
| Antihypertensive medication | 2,880 (11.43) | 596 (22.34) |
| Atrial fibrillation | 211 (0.84) | 44 (1.65) |
| Atypical antipsychotic | 70 (0.28) | 8 (0.30) |
| Chronic kidney disease (stages 3-5) | 13 (0.05) | 0 (0.00) |
| Diabetes | 537 (2.13) | 116 (4.35) |
| Erectile dysfunction | 127 (0.50) | 29 (1.09) |
| Ethnicity: Black | 429 (1.70) | 24 (0.90) |
| Ethnicity: Other | 1,004 (3.98) | 94 (3.52) |
| Family history of coronary artery disease | 8,856 (35.14) | 1,189 (44.57) |
| Migraine | 369 (1.46) | 40 (1.50) |
| Rheumatoid arthritis | 164 (0.65) | 30 (1.12) |
| Severe mental health disorder | 108 (0.43) | 17 (0.64) |
| Smoker: current | 2,753 (10.92) | 392 (14.69) |
| Smoker: former | 8,993 (35.68) | 1,095 (41.04) |
| Systemic lupus erythematosus | 8 (0.03) | 3 (0.11) |
| Systemic steroid | 172 (0.68) | 31 (1.16) |

**(B)**

| **Variable** | **Non-cases** | **Cases** |
| --- | --- | --- |
| *N* | 38,938 | 2,044 |
|  |  | |
|  | **Mean (s.d.)** | |
| Age (years) | 55.10 (7.95) | 60.00 (6.90) |
| Body mass index (kg/m2) | 26.61 (4.92) | 27.86 (5.39) |
| Systolic blood pressure: mean (mmHg) | 133.64 (18.80) | 142.10 (19.92) |
| Systolic blood pressure: s.d. (mmHg) | 5.37 (4.41) | 6.06 (4.90) |
| Townsend deprivation index | -1.46 (2.98) | -1.21 (3.10) |
| Alanine aminotransferase (U/L) | 19.46 (11.34) | 21.14 (13.99) |
| Albumin (g/L) | 44.96 (2.58) | 44.45 (2.63) |
| Alkaline phosphatase (U/L) | 83.11 (26.13) | 92.16 (28.07) |
| Apolipoprotein A1 (g/L) | 1.64 (0.27) | 1.60 (0.27) |
| Apolipoprotein B (g/L) | 1.05 (0.23) | 1.13 (0.24) |
| Aspartate aminotransferase (U/L) | 23.98 (8.58) | 25.47 (12.62) |
| Calcium (mmol/L) | 2.38 (0.10) | 2.39 (0.10) |
| Cholesterol (mmol/L) | 230.86 (41.69) | 241.86 (42.14) |
| C-reactive protein (mg/L) | 2.55 (4.17) | 3.61 (5.17) |
| Creatinine (µmol/L) | 63.96 (11.01) | 64.66 (11.75) |
| Cystatin C (mg/L) | 0.86 (0.14) | 0.93 (0.16) |
| Direct bilirubin (µmol/L) | 1.53 (0.66) | 1.44 (0.50) |
| Gamma glutamyltransferase (U/L) | 28.38 (29.29) | 34.00 (35.90) |
| Glucose (mmol/L) | 4.97 (0.82) | 5.12 (1.13) |
| Glycated hemoglobin (mmol/mol) | 34.93 (4.65) | 36.43 (6.04) |
| High-density lipoprotein cholesterol (mmol/L) | 62.38 (14.38) | 59.08 (13.96) |
| Insulin-like growth factor 1 (nmol/L) | 21.29 (5.67) | 19.77 (5.64) |
| Lipoprotein(a) (g/L) | 44.56 (48.76) | 48.55 (50.40) |
| Low density cholesterol (mmol/L) | 3.71 (0.84) | 3.98 (0.84) |
| Phosphate (mmol/L) | 1.19 (0.15) | 1.19 (0.15) |
| Sex hormone binding globulin (nmol/L) | 64.20 (31.44) | 61.62 (31.37) |
| Testosterone (nmol/L) | 1.11 (0.62) | 1.16 (0.77) |
| Total bilirubin (µmol/L) | 8.18 (3.73) | 7.75 (3.15) |
| Total protein (g/L) | 72.36 (4.08) | 72.43 (4.31) |
| Triglycerides (mmol/L) | 1.48 (0.81) | 1.75 (0.88) |
| Urate (µmol/L) | 264.79 (62.37) | 283.33 (68.64) |
| Urea (mmol/L) | 5.14 (1.23) | 5.41 (1.30) |
| Vitamin D (µg) | 48.47 (20.69) | 47.12 (19.96) |
| Basophil count (e9 cells/L) | 0.04 (0.05) | 0.04 (0.07) |
| Eosinophil count (e9 cells/L) | 0.16 (0.13) | 0.17 (0.12) |
| Hematocrit (%) | 39.14 (2.76) | 39.56 (2.78) |
| Hemoglobin concentration (g/dL) | 13.46 (0.95) | 13.61 (0.98) |
| High light scatter reticulocyte count (e12 cells/L) | 0.02 (0.01) | 0.02 (0.01) |
| Immature reticulocyte fraction | 0.29 (0.06) | 0.29 (0.06) |
| Lymphocyte count (e9 cells/L) | 1.99 (0.98) | 2.05 (0.66) |
| Mean corpuscular hemoglobin (pg) | 31.30 (1.88) | 31.29 (1.92) |
| Mean corpuscular hemoglobin concentration (g/dL) | 34.41 (0.98) | 34.41 (0.91) |
| Mean corpuscular volume (fL) | 90.92 (4.61) | 90.91 (4.95) |
| Mean platelet volume (fL) | 9.37 (1.09) | 9.33 (1.07) |
| Mean reticulocyte volume (fL) | 105.59 (7.68) | 105.82 (8.16) |
| Mean sphered cell volume (fL) | 83.08 (5.24) | 83.15 (5.66) |
| Monocyte count (e9 cells/L) | 0.43 (0.19) | 0.46 (0.21) |
| Neutrophil count (e9 cells/L) | 4.14 (1.36) | 4.36 (1.48) |
| Nucleated red blood cell count (e9 cells/L) | 0.02 (0.05) | 0.02 (0.02) |
| Platelet count (e9 cells/L) | 266.32 (59.70) | 271.35 (61.10) |
| Platelet crit (%) | 0.25 (0.05) | 0.25 (0.05) |
| Platelet distribution width (%) | 16.41 (0.50) | 16.43 (0.49) |
| Red blood cell count (e12 cells/L) | 4.31 (0.33) | 4.36 (0.33) |
| Red blood cell distribution width (%) | 13.50 (1.04) | 13.60 (1.04) |
| Reticulocyte count (e9 cells/L) | 0.06 (0.03) | 0.06 (0.02) |
| White blood cell count (e9 cells/L) | 6.76 (1.90) | 7.08 (1.81) |
| 3-Hydroxybutyrate (mmol/l) | 0.06 (0.06) | 0.06 (0.06) |
| Acetate (mmol/l) | 0.02 (0.01) | 0.01 (0.02) |
| Acetoacetate (mmol/l) | 0.01 (0.01) | 0.01 (0.01) |
| Acetone (mmol/l) | 0.01 (0.01) | 0.01 (0.01) |
| Alanine (mmol/l) | 0.29 (0.07) | 0.29 (0.07) |
| Citrate (mmol/l) | 0.06 (0.01) | 0.06 (0.01) |
| Fatty acids: degree of unsaturation | 1.38 (0.07) | 1.37 (0.08) |
| Glutamine (mmol/l) | 0.52 (0.08) | 0.53 (0.08) |
| Glycine (mmol/l) | 0.19 (0.07) | 0.18 (0.07) |
| Glycoprotein acetyls (mmol/l) | 0.78 (0.12) | 0.83 (0.12) |
| Histidine (mmol/l) | 0.06 (0.01) | 0.06 (0.01) |
| Lactate (mmol/l) | 3.71 (1.07) | 3.73 (1.07) |
| Omega-3 fatty acids (mmol/l) | 0.55 (0.22) | 0.58 (0.23) |
| Phenylalanine (mmol/l) | 0.04 (0.01) | 0.05 (0.01) |
| Pyruvate (mmol/l) | 0.08 (0.03) | 0.08 (0.03) |
| Total branched-chain amino acids (mmol/l) | 0.33 (0.08) | 0.34 (0.08) |
| Total cholines (mmol/l) | 2.67 (0.39) | 2.71 (0.40) |
| Tyrosine (mmol/l) | 0.06 (0.01) | 0.06 (0.01) |
| Polygenic risk score | -0.00 (0.79) | 0.17 (0.82) |
|  | **Prevalence of “yes” (%)** | |
| Antihypertensive medication | 4,504 (11.57) | 505 (24.71) |
| Atrial fibrillation | 131 (0.34) | 30 (1.47) |
| Atypical antipsychotic | 73 (0.19) | 3 (0.15) |
| Chronic kidney disease (stages 3-5) | 9 (0.02) | 1 (0.05) |
| Diabetes | 478 (1.23) | 71 (3.47) |
| Ethnicity: Black | 711 (1.83) | 26 (1.27) |
| Ethnicity: Other | 1,316 (3.38) | 59 (2.89) |
| Family history of coronary artery disease | 16,527 (42.44) | 1,121 (54.84) |
| Migraine | 1,784 (4.58) | 111 (5.43) |
| Rheumatoid arthritis | 505 (1.30) | 51 (2.50) |
| Severe mental health disorder | 161 (0.41) | 14 (0.68) |
| Smoker: current | 3,054 (7.84) | 253 (12.38) |
| Smoker: former | 12,353 (31.72) | 715 (34.98) |
| Systemic lupus erythematosus | 82 (0.21) | 10 (0.49) |
| Systemic steroid | 275 (0.71) | 42 (2.05) |

**Supplementary Table 5.** Hazard ratios of covariates selected by LASSO stability selection in Cox models for cardiovascular disease incidence, fit on training data.

|  | Men | Women |
| --- | --- | --- |
| Age | 1.57 | 1.61 |
| *Albumin | 0.91 | 0.91 |
| Antihypertensive medication (Y/N) | 1.08 | 1.11 |
| *Apolipoprotein A1 | 0.9 | - |
| *Apolipoprotein B | 1.17 | 1.08 |
| Atrial fibrillation (Y/N) | 1.03 | 1.06 |
| *C-reactive protein | 1.1 | 1.12 |
| Current smoking status | 1.09 | 1.2 |
| *Cystatin C | 1.1 | 1.2 |
| Family history of coronary artery disease (Y/N) | 1.14 | 1.13 |
| *Glycated hemoglobin | 1.06 | 1.04 |
| *Lipoprotein(a) | 1.12 | - |
| Polygenic Risk Score | 1.22 | 1.17 |
| Systolic blood pressure: mean | 1.19 | 1.19 |
| Townsend deprivation index | 1.07 | - |
| *Triglycerides | - | 1.06 |
| *White blood cell count | 1.06 | - |

*Variables were log-transformed

**Supplementary Table 6.** Reclassification of cardiovascular disease cases and non-cases with nested models, i.e., comparing Cox survival models with and without LASSO stability selected variables alongside log hazards from respective validated risk prediction algorithms: (A) at 7.5% 10-year risk threshold for pooled cohort equations (PCE) and (B) at 10% 10-year risk threshold for QRISK3. Sensitivity and specificity are shown at the relevant risk thresholds.

**A** Reclassification of cardiovascular disease cases and non-cases for PCE

**Men**

|  | | | **LASSO stability selection** | | |
| --- | --- | --- | --- | --- | --- |
|  |  |  | **Predicted 10-year risk (%)** | | **Reclassified (%)** |
| **PCE**  **Predicted 10-year risk (%)** |  |  | **<7.5** | **≥7.5** |  |
|  | **Cases** | **<7.5** | 871 | 376 | 30.2 |
|  |  | **≥7.5** | 292 | 2,032 | 12.6 |
|  | **Non-cases** | **<7.5** | 17,404 | 2,333 | 11.8 |
|  |  | **≥7.5** | 3,151 | 10,060 | 23.9 |

|  | **LASSO** | **PCE** |
| --- | --- | --- |
| **Sensitivity (at 7.5% 10-year risk)** | 67.43% | 65.08% |
| **Specificity (at 7.5% 10-year risk)** | 62.39% | 59.90% |

**Women**

|  | | | **LASSO stability selection** | | |
| --- | --- | --- | --- | --- | --- |
|  |  |  | **Predicted 10-year risk (%)** | | **Reclassified (%)** |
| **PCE**  **Predicted 10-year risk (%)** |  |  | **<7.5** | **≥7.5** |  |
|  | **Cases** | **<7.5** | 1,733 | 346 | 16.6 |
|  |  | **≥7.5** | 143 | 512 | 21.8 |
|  | **Non-cases** | **<7.5** | 44,528 | 2,406 | 5.1 |
|  |  | **≥7.5** | 1,815 | 3,309 | 35.4 |

|  | **LASSO** | **PCE** |
| --- | --- | --- |
| **Sensitivity (at 7.5% 10-year risk)** | 31.38% | 23.96% |
| **Specificity (at 7.5% 10-year risk)** | 89.02% | 90.16% |

**B** Reclassification of cardiovascular disease cases and non-cases for QRISK3

**Men**

|  | | | **LASSO stability selection** | | |
| --- | --- | --- | --- | --- | --- |
|  |  |  | **Predicted 10-year risk (%)** | | **Reclassified (%)** |
| **QRISK3**  **Predicted 10-year risk (%)** |  |  | **<10** | **≥10** |  |
|  | **Cases** | **<10** | 1403 | 252 | 15.2 |
|  |  | **≥10** | 224 | 1692 | 11.7 |
|  | **Non-cases** | **<10** | 22450 | 1480 | 6.2 |
|  |  | **≥10** | 2017 | 7001 | 22.4 |

|  | **LASSO** | **QRISK3** |
| --- | --- | --- |
| **Sensitivity (at 10% 10-year risk)** | 54.44% | 53.65% |
| **Specificity (at 10% 10-year risk)** | 74.26% | 72.63% |

**Women**

|  | | | **LASSO stability selection** | | |
| --- | --- | --- | --- | --- | --- |
|  |  |  | **Predicted 10-year risk (%)** | | **Reclassified (%)** |
| **QRISK3**  **Predicted 10-year risk (%)** |  |  | **<10** | **≥10** |  |
|  | **Cases** | **<10** | 2102 | 174 | 7.6 |
|  |  | **≥10** | 80 | 378 | 17.5 |
|  | **Non-cases** | **<10** | 48609 | 1018 | 2.1 |
|  |  | **≥10** | 679 | 1752 | 27.9 |

|  | **LASSO** | **QRISK3** |
| --- | --- | --- |
| **Sensitivity (at 10% 10-year risk)** | 20.19% | 16.75% |
| **Specificity (at 10% 10-year risk)** | 94.68% | 95.33% |

**Supplementary Figure 1.** Calibration plots showing predicted and observed 10-year risk of cardiovascular disease by decile of log hazard using pooled cohort equations (blue) and QRISK3 (red) in men (A-D) and women (E-H). Probabilities computed in training (A, E: non-recalibrated; B, F: recalibrated) and test sets (C, G: non-recalibrated; D, H: recalibrated). Pooled cohort equations and QRISK3 were recalibrated by using computed log hazards as predictor in Cox models fitted on sex-stratified training sets. Slopes (β) are reported.

**Supplementary Figure 2.** Heatmap of Pearson’s correlations between biochemistry (green), PCE/QRISK3 (blue), genetic (brown), hematology (red) and Nightingale (purple) markers. Measured levels of biochemistry, hematology and Nightingale biomarkers were log-transformed.

**Supplementary Figure 3.** C-statistics in test data using Cox models sequentially including predictors in descending order of selection proportion in men (A) and women (B). Results are presented for all variables, and the vertical red dashed line indicates the model including all stably selected variables. C-statistics for recalibrated PCE (black horizontal dashed line) and QRISK (orange horizontal dashed line) are also reported. Color code: Variables in either PCE or QRISK3 – blue; biochemistry – green; hematology – red; polygenic risk score – brown.

**Supplementary Figure 4.**  Scatter plots comparing the LASSO stability selection proportions from the base model and model where either pooled cohort equations (PCE, top panel: A in men and B in women, respectively) or QRISK3 (bottom panel: C in men and D in women, respectively) log hazards are included in place of their constituent variables.

**Supplementary Figure 5.** LASSO stability selection proportions from the model excluding (X-axis) and including (Y-axis) metabolomic data in (N=11,149) men (A) and (N=16,392) women with metabolomic data available (B). Calibrated thresholds in selection proportion are shown as dashed dark-red lines. In (A) and (B), variables to the right of the dashed red vertical line were selected in the base model, and variables above the horizontal dashed red line were selected in the model including metabolomic data. The horizontal dotted purple lines indicate the selection proportions for each metabolomic variable. For clarity, the per-feature selection proportions are only reported for the three most frequently selected metabolites in each plot.

**Supplementary Figure 6.** C statistics in test data using Cox models sequentially including predictors in descending order of selection proportion from the base model (A, B in men and women, respectively) and the model further including metabolomic variables (C, D in men and women, respectively). Results are presented for all variables, and the vertical red dashed line indicates the model including all stably selected variables. C-statistics for recalibrated PCE (black horizontal dashed line) and QRISK (orange horizontal dashed line) are also reported. Color code: Variables in either PCE or QRISK3 – blue; biochemistry – green; hematology – red; polygenic risk score – brown.

**Supplementary Figure 7.** Receiver operating characteristic (ROC) curves from logistic models for incident CVD, excluding (top panel: A and B in men and women, respectively) and including (bottom panel: C and D in men and women, respectively) NMR-derived metabolomic variables. Results from logistic models including PCE constituents (blue line), QRISK3 (green) or sex-specific LASSO stably selected variables (red line) are shown, with mean area under the curve (AUC) and 95% confidence intervals.

**Supplementary Figure 8**. Per feature selection proportion for each of the 100 stability selection models fitted and calibrated on independent randomly sampled variable selection sets. For each predictor, we plot the (N=100) estimated selection proportion and report the mean selection proportion across the 100 models. Results are presented for models excluding metabolomic data (A and B in men and women, respectively) and including metabolomic data (C and D in men and women, respectively).

**Methods**

*Variable definitions*

Self-reported ethnicity was defined from UK Biobank field 21000 to reproduce ethnicity categories used in pooled cohort equations (White, Black, Other). Pooled cohort equations compute cardiovascular risk using the same weightings for White and Other ethnicity as well as for individuals not entering their ethnicity, with Black ethnicity having a different set of weightings. Black ethnicity included codes 4, 2001, 2002, 4001, 4002 and 4003. White ethnicity included codes 1, 1001, 1002 and 1003. If UK Biobank data field 21000 had missing data, was answered “Do not know” or “Prefer not to answer”, ethnicity was coded as Other.

Diabetes in pooled cohort equations is a binary variable (Y/N). Here, “yes” included any type of diabetes mellitus according to:

- self-report (UK Biobank field 20002 – codes: 1220, 1222, 1223)
- self-reported diabetic medication use (UK Biobank field 20003 – codes: 1140868902, 1140874646, 1140874674, 1140874718, 1140874744, 1140883066, 1140884600, 1141152590, 1141157284, 1141168660, 1141171646, 1141173882, 1141189090)
- hospitalization prior to enrolment (ICD-9 codes: 25000, 25010, 25020, 25030, 25040, 25050, 25060, 25070, 25080, 25090, 25002, 25012, 25022, 25032, 25042, 25052, 25062, 25072, 25082, 25092, 25001, 25011, 25021, 25031, 25041, 25051, 25061, 25071, 25081, 25091, 25003, 25013, 25023, 25033, 25043, 25053, 25063, 25073, 25083, 25093. ICD-10 codes: E10, E11, O230, O231.
- glycated hemoglobin at baseline was not used to define diabetes to avoid capturing unknown cases.

Smoking status (never, former, current) was defined using UK Biobank fields 20116, 22508, 3456, 1239, 22506, 22508 and 3446.

*Variable filtering*

Circulating levels of 168 metabolomic biomarkers, including fatty acids, cholesterol, lipoproteins, phospholipids, amino acids and ketone bodies, were estimated by deconvolution of an NMR spectral profile in a subset of N=117,413 UK Biobank participants at baseline (N=73,345 in our study population). For the N=5 biomarkers (albumin, apolipoprotein A1, apolipoprotein B, creatinine and glucose) available on both the NMR and biochemistry platforms, only the directly measured biochemistry levels were retained for analysis. All lipid-related markers (N=125) and a set of non-lipid metabolomic markers (N=18) were excluded due to strong correlations with the directly measured biochemistry variables ($\rho$>0.7). The total concentration of branched-chain amino acids was used instead of its individual components (Valine, Leucine and Isoleucine) which were strongly correlated ($\rho$>0.8). Similarly, phosphatidylcholines and phosphoglycerides, which were both strongly correlated with total cholines ($\rho$>0.9) were excluded. After filtering, a total of 18 metabolomic features, with low to moderate correlations with each other and with biochemistry and hematology markers, remained for analysis (Supplementary Figure 1). Complete data for the 18 retained metabolomic markers were available for N=68,855 participants.

*Recalibration of PCE and QRISK3*

Pooled cohort equations and QRISK3 were recalibrated in the training set in men and women separately to correct for possible over or under-estimation of CVD risk in UK Biobank and to allow for fair comparison between models. Recalibration was achieved by fitting predicted log hazards from published PCE and QRISK3 algorithms, respectively, as a Cox model covariate, with baseline survival function estimated to give the intercept. Calibration was assessed graphically by plotting observed probability of CVD (Kaplan-Meier estimate) against mean predicted probability within decile of predicted probabilities. We calculated the calibration slope to assess possible differences between observed and expected probabilities (Supplementary Figure 2).

*Variable contribution to Cox model performance*

To evaluate the contribution of each stably-selected variable to model performance, we ran a series of unpenalized Cox models, sequentially adding predictors in descending order of selection proportion. These models were fitted on the test set to estimate the mean and 95% confidence interval of the C-statistics.

*LASSO stability selection sensitivity analysis*

We ran our stability selection approach on 100 randomly sampled variable selection sets. For each training set, we ran the same LASSO-regularized Cox survival models calibrated via stability using (N=1,000) subsamples. Each of the 100 models returned a calibrated per-feature selection proportion, which was examined to infer the reproducibility of our results and of the variables selected using our approach.
